# The effect of maternal vitamin D deficiency during pregnancy on body fat and adipogenesis in rat offspring

**DOI:** 10.1038/s41598-017-18770-4

**Published:** 2018-01-10

**Authors:** Juan Wen, Qin Hong, Xingyun Wang, Lijun Zhu, Tianqi Wu, Pengfei Xu, Ziyi Fu, Lianghui You, Xing Wang, Chenbo Ji, Xirong Guo

**Affiliations:** 1 Nanjing Maternity and Child Health Care Institute, Nanjing Maternity and Child Health Care Hospital, Obstetrics and Gynecology Hospital Affiliated to Nanjing Medical University, Nanjing, 210004 China; 2 Department of Children Health Care, Nanjing Maternity and Child Health Care Hospital, Obstetrics and Gynecology Hospital Affiliated to Nanjing Medical University, Nanjing, 210004 China; 3 State key Laboratory of Reproductive Medicine, Nanjing Maternity and Child Health Care Hospital, Obstetrics and Gynecology Hospital Affiliated to Nanjing Medical University, Nanjing, 210004 China; 4 Institute of Pediatrics, Nanjing Medical University, Nanjing, 210029 China

## Abstract

To evaluate the effects of maternal vitamin D deficiency on body fat and adipogenesis in offspring rats, and explore the potential mechanism, we constructed a vitamin D deficient rat model and performed metabolic activity evaluation, body fat monitoring, biochemical analysis, adipogenesis assay, methylation microarray and RNA-seq for their offspring rats. We found the weight of vitamin D deficient (VDD) offspring was gradually higher than that of control (CLT) offspring, and the difference was significant since 10 weeks old. When compared with CTL offspring, the 24 h heat production, peak blood glucose, adipose tissue volume and blood lipid indexes were significantly increased in VDD offspring at 14 weeks old. Moreover, a significant increase in proliferation rate and number of lipid droplets for pre-adipocytes was also observed in VDD offspring group. DNA methylation profiling showed that compared to CTL group, 608 promoters and 204 CpG islands were differentially methylated in the VDD group, involving 305 genes. When combined with the results of RNA-seq, 141 genes of the methylated genes were differentially expressed. In conclusion, vitamin D deficiency during pregnancy may promote the proliferation and differentiation of pre-adipocytes, which may be associated with methylation alterations of genes, ultimately leading to offspring obesity.

## Introduction

Overweight and obesity is an important and increasingly prevalent public health problem worldwide^1,2^. When compared to non-obese people, obese people are at greater risk of diabetes, hypertension, dyslipidemia, and other cardiovascular disease^3^. Thus, obesity and its serious comorbidities exert a heavy toll in both human and economic terms^3^. The occurrence and development of obesity is a very complicated process involving heredity, environment, diet structure and many other factors. There is more and more evidence that non-proper nutrient in early life (including during pregnancy and lactation) may directly contribute to the epidemic of adiposity in childhood, adolescence and adulthood^4,5^. Due to the presence of an abundance of vitamin D receptors (VDR) on adipocytes, the association between vitamin D and obesity has been the focus of attention^6,7^.

Vitamin D is a fat-soluble secosteroid which is transported to the liver for hydroxylation to 25-hydroxyvitamin D [25(OH)D], the main circulating form of vitamin D and the best measure of vitamin D status. The second hydroxylation to the active form 1,25-hydroxyvitamin D [1,25(OH)D] occurs mostly in the kidneys. Then, vitamin D binds to VDR and participates in a wide array of activities including calcium and phosphorus metabolism, embryonic tissue development and mature, immune function, cellular growth and differentiation, and glucose metabolism^8–10^. Many studies have suggested that the vitamin D level is significantly lower in obese populations than in non-obese populations. A survey of the 1958 British birth cohort from 7,198 Caucasian subjects showed that serum 25(OH)D concentrations decreased with increasing body mass index (BMI)^11^. Even though in the non-obese populations, BMI and percentage body fat content were inversely related to the serum 25(OH)D concentrations^12,13^. Moreover, women and children with vitamin D deficiency had higher rates of obesity than those with non-deficient vitamin D level^14^. In a randomized controlled trial for calcium plus vitamin D supplementation, a significantly greater decrease in fat mass loss was observed in the calcium plus vitamin D supplementation group than in the control group, although there was no significant difference in body weight change between groups^15^.

Vitamin D deficiency in pregnant women has become a very common phenomenon^16,17^. An observational study including 977 pregnant women showed lower maternal vitamin D status was associated with greater fat mass at ages 4 and 6 y^18^. And a Spain cohort also yielded similar results and concluded that vitamin D deficiency in pregnancy may increase the risk of prenatal and early postnatal overweight in offspring^19^. In our previous large cohort study, we found women with 25(OH)D < 37.5 nmol/L had infants with higher birth weight in a linear regression model, and women with 25(OH)D < 50.0 nmol/L had increased risk of macrosomia, when compared with women with 25(OH)D concentrations from 50.0 to 74.9 nmol/L^20^. Thus, the effects of maternal vitamin D deficiency on birth weight or body fat of offspring could not be ignored. Furthermore, evidence has shown that the effects of maternal nutrition on offspring obesity may be related with epigenetic mechanisms^21,22^. And data are now emerging suggesting that alterations in DNA methylation is central to the processes of developmental plasticity and also support the relationships between early life effects and later metabolic status^23,24^. Therefore, we constructed a vitamin D deficient rat model and performed metabolic activity evaluation, body fat monitoring, biochemical analysis, adipogenesis assay, methylation microarray and RNA-seq for their offspring rats to evaluate the effects of vitamin D deficiency during pregnancy on body fat and adipogenesis in offspring rats.

## Results

**Figure 1:**
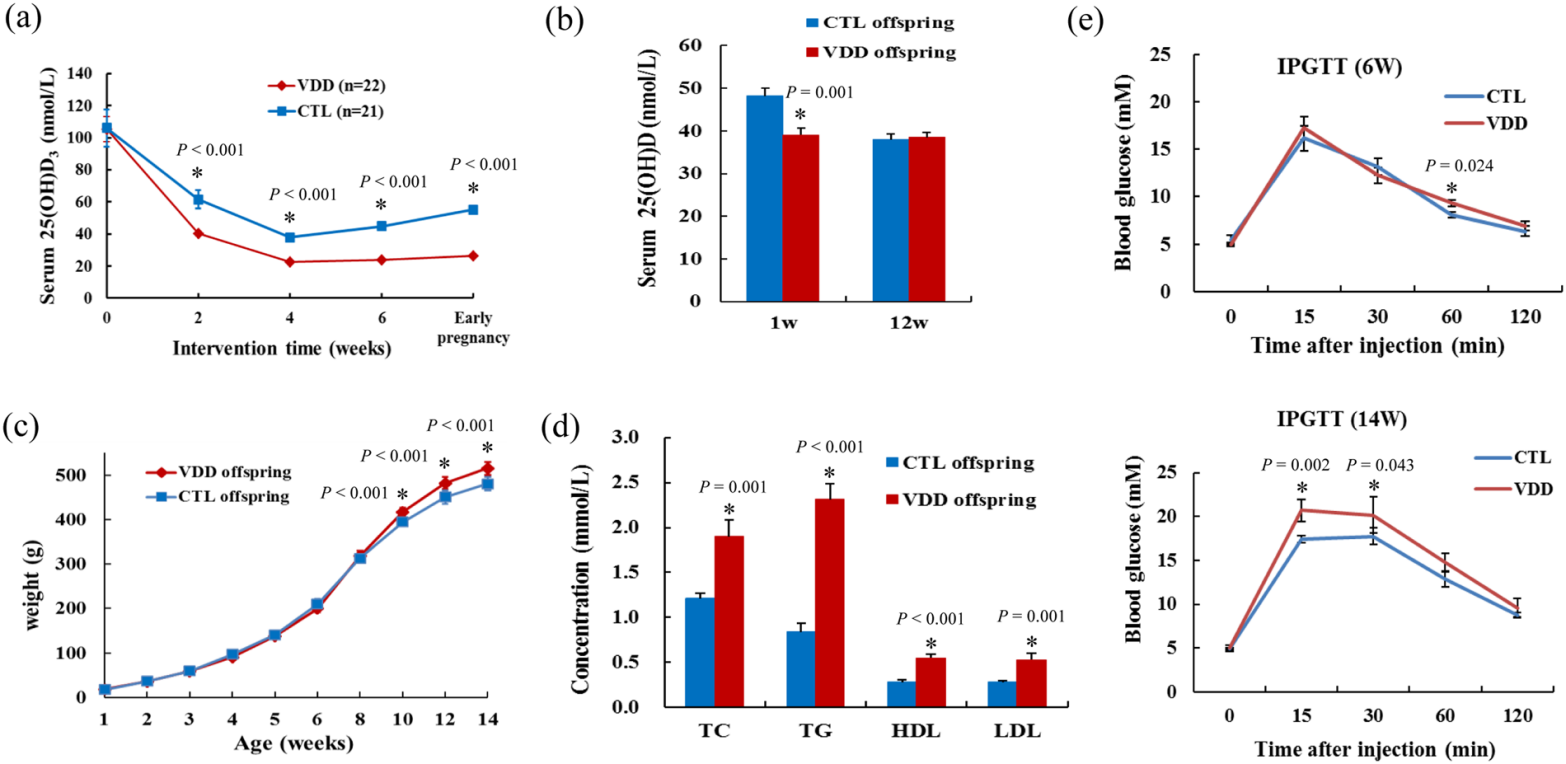
The vitamin D deficient rat model was constructed successfully and the effects of maternal vitamin D deficiency on weight, blood lipid indexes and blood glucose levels for offspring. (**a**,**b**) The 25(OH)D concentrations of the VDD and CLT dams and their offspring. (**c**) The weight of the VDD and CLT offspring. (**d**) The plasma concentrations of TC, TG, HDL, and LDL of VDD and CTL offspring. (**e**) Blood glucose levels after the glucose injection (at 0, 15, 30, 60, 120 min) of VDD and CTL offspring at six and fourteen week of age. VDD, vitamin D deficient group; CTL, control; TC, total cholesterol; TG, triglycerides; HDL, high density lipoprotein; LDL, low density lipoprotein; Data are represented as mean ± SE; *P < 0.05.

Removal of dietary vitamin D prior to conception and throughout gestation resulted in total depletion of 25(OH)D in the vitamin D deficient (VDD) dams, when compared with the control (CLT) dams (Fig. 1a). There was no significant difference in weight and pup numbers between the CTL and VDD dams. Although the 25(OH)D concentrations of the VDD and CLT offspring at 1 week old largely reflected the levels in their respective dams (Fig. 1b), the VDD and CLT offspring in birth weight did not exist significant difference (*P* > 0.05). However, we found the weight of VDD offspring was gradually higher than that of CTL offspring, and the difference was significant since the offspring at 10 weeks old (Fig. 1c).

Biochemical analysis for offspring at 14 weeks old showed that the plasma concentrations of TC, TG, HDL, and LDL were significantly increased in VDD offspring when compared with CTL offspring (*P* < 0.05) (Fig. 1d). We also measured blood glucose for VDD and CTL offspring at each week of age, and found the blood glucose of VDD offspring was significantly higher than that of CTL offspring at 60 min after glucose injection at 6 weeks old, and at 15 and 30 min after glucose injection at 14 weeks old. However, the blood glucose of the two groups reached the same level at 120 min after injection (Fig. 1e).

**Figure 2:**
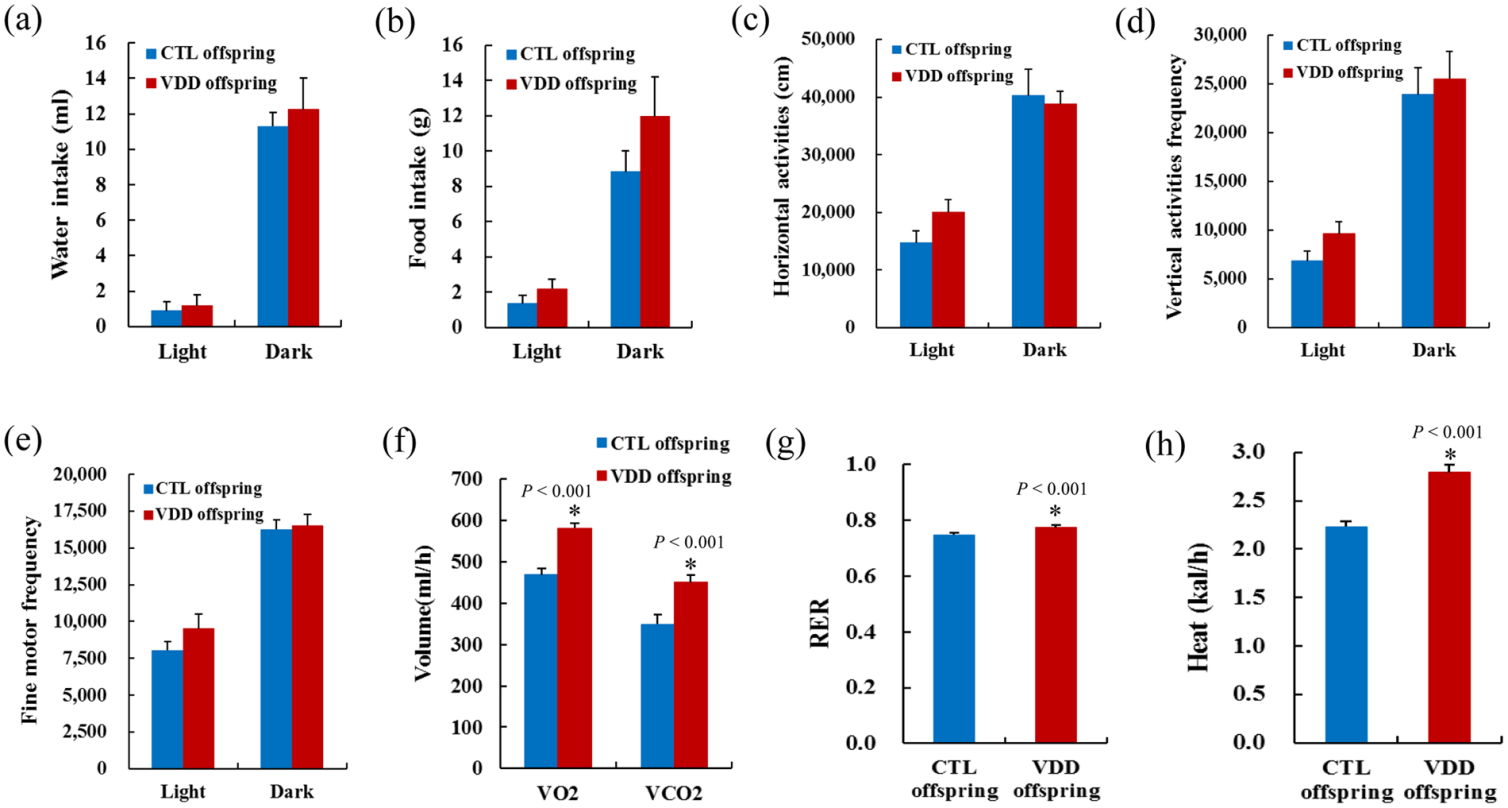
The feeding behavior, locomotor activity and energy expenditure of the VDD and CLT offspring. (**a**–**e**) Feeding behavior (water and food intake) and locomotor activity (horizontal, vertical and fine motor activity) of VDD and CTL offspring. (**f**–**h**) The 24 h VO2, VCO2, RER and heat production of VDD and CTL offspring. VDD, vitamin D deficient group; CTL, control; VO_2_, oxygen consumption; VCO_2_, carbon dioxide production; RER, respiratory exchange rate; Data are represented as mean ± SE; *P < 0.05.

For the offspring at 14 weeks old, feeding behavior, locomotor activity, and the energy expenditure were measured using the PhenoMaster system. Removal of dietary vitamin D prior to conception and throughout gestation did not cause any alterations of feeding behavior (water and food intake) and locomotor activity (horizontal, vertical and fine motor activity) (*P* > 0.05) (Fig. 2a–e). However, the 24 h VO_2_, VCO_2_, RER and heat production of VDD offspring were significantly higher than that of CLT offspring (*P* < 0.05) (Fig. 2f–h).

**Figure 3:**
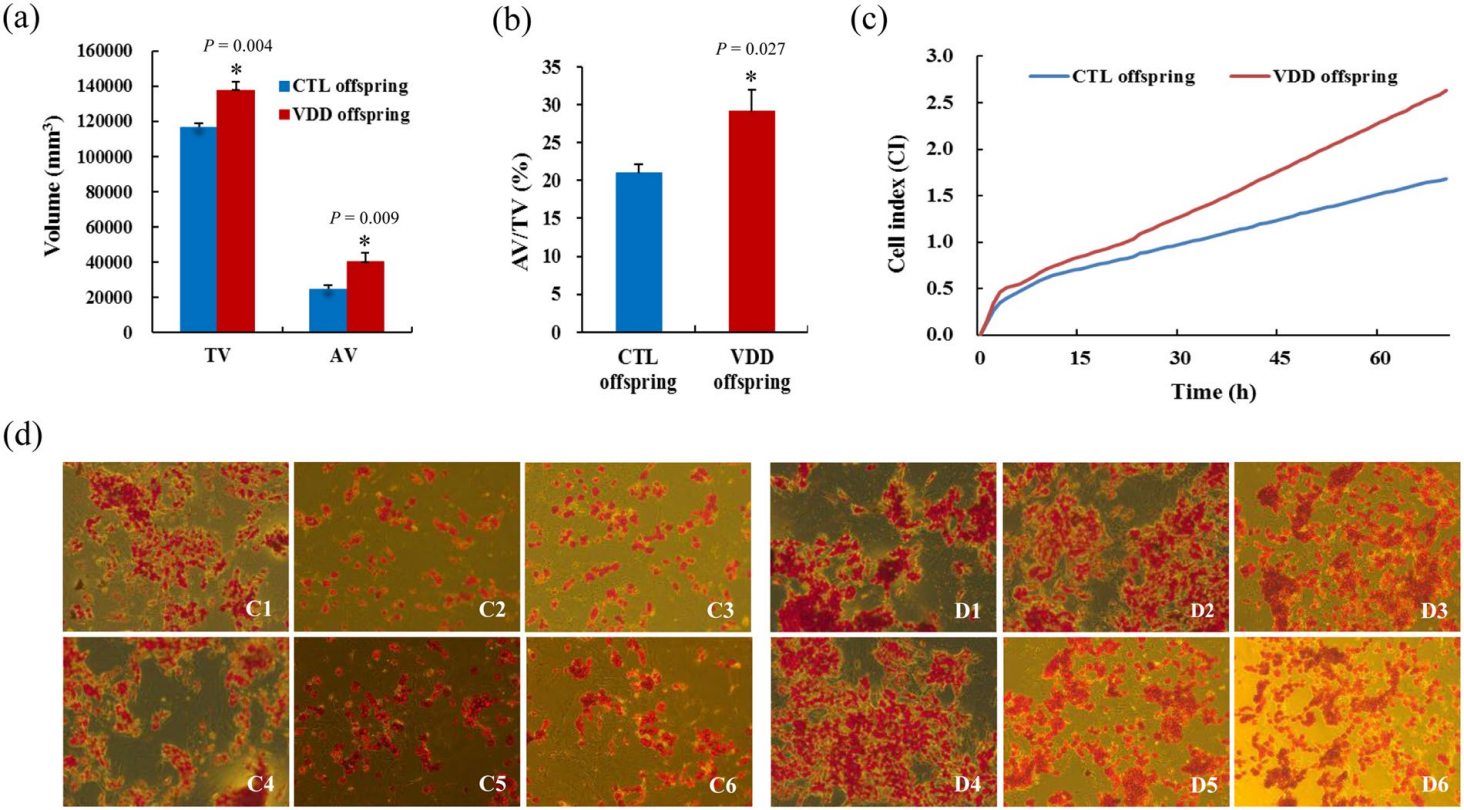
The effects of vitamin D deficiency on adipose tissue volume and adipogenesis. (**a**,**b**) The TV, AV, AV/TV of VDD and CTL offspring. (**c**,**d**) xCELLigence assays and oil red O staining showed a significant increase in proliferation rate and number of lipid droplets of pre-adipocytes in VDD group compared with CTL group. VDD, vitamin D deficient group; CTL, control; TV, total volume; AV, adipose tissue volume; C1–C6, six samples from CTL offspring; D1–D6, six samples from VDD offspring; Data are represented as mean ± SE; *P < 0.05.

**Figure 4:**
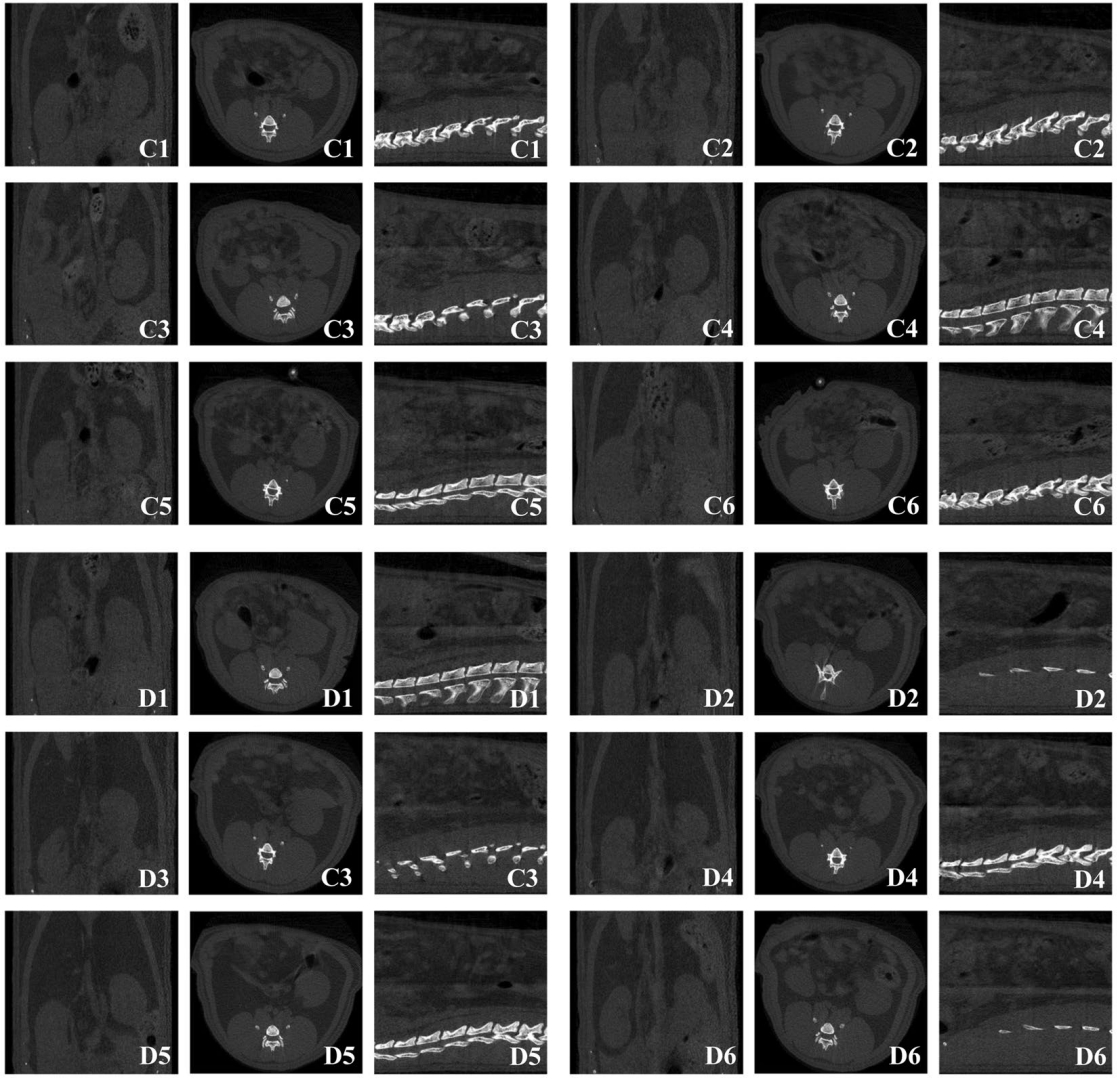
Transverse micro-CT images of the waist of offspring rats for body fat monitoring. C1–C6, six samples from CTL offspring; D1–D6, six samples from VDD offspring.

Further body fat monitoring for offspring at 14 weeks old showed that TV, AV and AV/TV were significantly increased in VDD offspring when compared with CTL offspring (*P* < 0.05) (Figs 3a,b and 4). To gain further insights into the effect of vitamin D deficiency on adipogenesis, xCELLigence assays and oil red O staining were conducted on the pre-adipocytes isolated from offspring retroperitoneal adipose tissue, and showed a significant increase in proliferation rate and number of lipid droplets in VDD group compared with CTL group (Fig. 3c,d).

**Figure 5:**
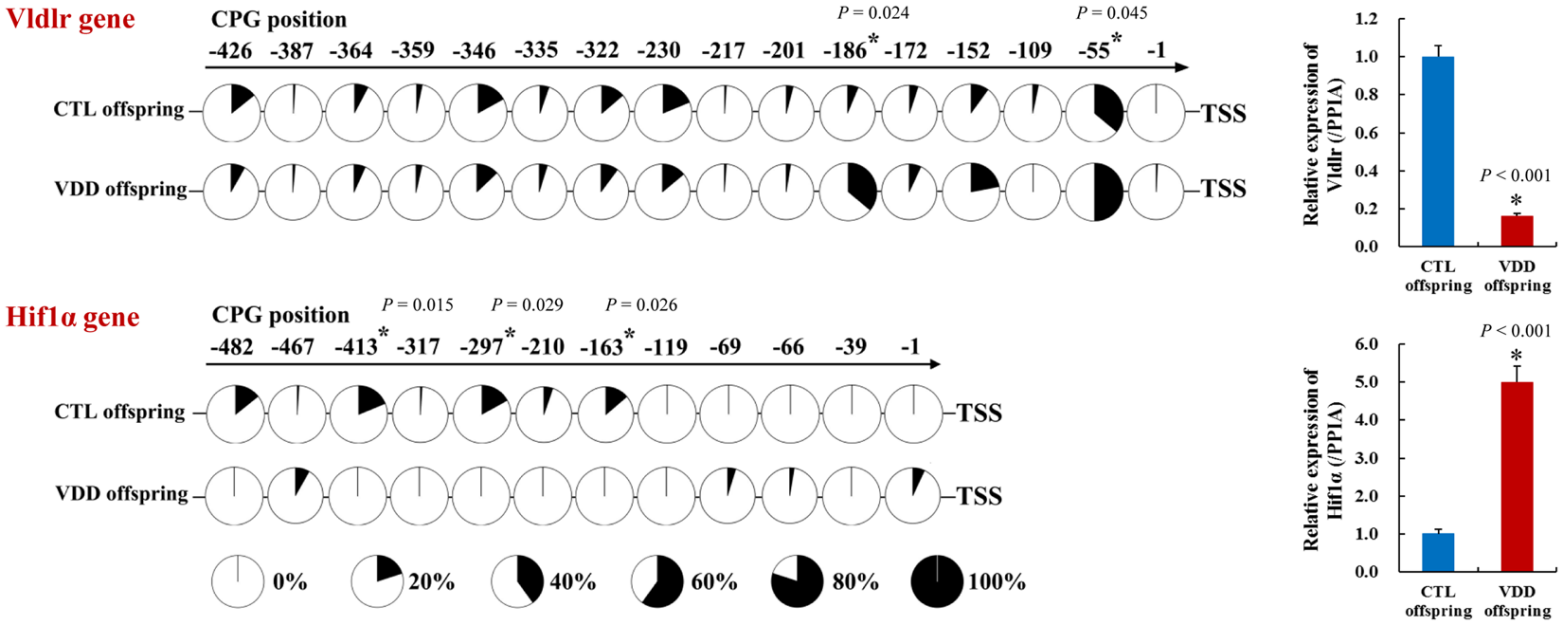
Methylation levels of *Vldlr* and *HIF1α* in promoter, and expression levels of *Vldlr* and *HIF1α*. VDD, vitamin D deficient group; CTL, control; Data are represented as mean ± SE; *P < 0.05.

To explore the potential mechanism for the effect of vitamin D deficiency on adipose adipogenesis, we performed a comprehensive DNA methylation profiling and RNA-seq analysis, and found that compared to CTL group, 608 promoters and 204 CpG islands (CGIs) were differentially methylated in the VDD group, involving 305 genes. When combined with the results of RNA-seq, 141 genes of the methylated genes were differentially expressed. Then we validated 7 obesity or lipid metabolism related genes (*Hif1a*, *Spon1*, *H19*, *Adrb2*, *Vldlr*, *Zfp36*, *Smad3*), and found that compared to CTL group, *Vldlr* was hypermethylated and low expressed, and *Hif1α* was demethylated and high expressed in the VDD group (Fig. 5). The *Vldlr* (Very low density lipoprotein receptor) gene encodes a lipoprotein receptor and plays important roles in VLDL-triglyceride metabolism and the reelin signaling pathway, whereas the *Hif1α* (Hypoxia inducible factor 1 alpha subunit) gene was involved in energy metabolism, angiogenesis, apoptosis, and other genes which could increase oxygen delivery or facilitate metabolic adaptation to hypoxia.

## Discussion

Our study comprehensively evaluated the effect of maternal vitamin D deficiency on the body weight, body fat, glucose and lipid metabolism and metabolic activity of the offspring, and found maternal vitamin D deficiency not only affected body weight, body fat, glucose and lipid metabolism in adulthood of offspring, but also affected proliferation and differentiation of the pre-adipocytes, suggesting non-proper nutrient in early life may directly contribute to the epidemic of adiposity in the future life. In order to better explain the mechanism behind the above phenomenon, we investigated the effect of maternal vitamin D deficiency on the alterations of DNA methylation in the adipose tissue of its offspring. We put forward the conclusion that vitamin D deficiency during pregnancy may promote the proliferation and differentiation of pre-adipocytes, which may be associated with methylation alterations of genes, such as *Vldlr* and *Hif1α*, ultimately leading to offspring obesity. The findings may provide some important clues for further mechanisms exploration.

In the animal experiments, we found the weight of VDD offspring was gradually higher than that of CTL offspring, but the birth weight between the VDD and CLT offspring did not exist significant difference. In a study for investigating the effects of maternal vitamin D deficiency on offspring kidney development, they found there were no differences in body mass at birth, but at six months, the vitamin D restricted groups were significantly heavier than their controls^25^, which was similar to the results of this study. More importantly, our results firstly suggested that maternal vitamin D deficiency could result in body fat increase of offspring. Large-scale, population-based studies showed that the negative correlation between serum vitamin D level and body fat was more prominent than that between vitamin D level and BMI^26^. Moreover, the plasma concentrations of TC, TG, HDL, and LDL were significantly increased in VDD offspring when compared with CTL offspring at 14 weeks old, suggesting lipid metabolism disorder was appeared in adulthood of offspring for maternal vitamin D deficiency. Population-based studies have shown that vitamin D level was closely related with lipid metabolism. For example, a randomized trial of 51 adults found a significant negative correlation between 25(OH)D and the ratio of LDL to HDL^27^. In our study, there was no significant difference in serum 25(OH)D between the CTL and VDD adult offspring, so we speculated that maternal vitamin D deficiency laid the foundations for lipid metabolism disorder in adulthood of offspring. Blood glucose is closely bound up with lipid metabolism. In the 2016 year’s European Association for the Study of Diabetes (EASD) meeting, vitamin D was indicated to improve insulin sensitivity and reduce the accumulation of fat in muscles though a mice model of diet-induced insulin resistance, which was constructed by Dr. Elisa Benetti and colleagues from the University of Turin, Italy (www.easdvirtualmeeting.org). And human studies have suggested a strong link between vitamin D deficiency and risk of diabetes regardless of age^28^. In addition, multiple studies and meta-analysis have shown that women with low 25(OH)D concentrations in pregnancy had significantly increased risks of gestational diabetes^29,30^. In summary, maternal vitamin D deficiency could result in changes of weight, body fat and metabolism in offspring. However, these changes were not related with vitamin D deficiency themselves. Thus, understanding the role of maternal nutrition in offspring outcomes merits further exploration.

The association of low vitamin D status with obesity has been well established^31^. It is reported that serum 25(OH)D < 50 nmol/L was significantly associated with new-onset obesity^32^. Recently, Wang *et al*. performed a genome-wide association study (GWAS) of the gut microbiota and discovered a significant association of the VDR gene (encoding vitamin D receptor) with gut microbial characteristics, which is essential for bile acid and fatty acid metabolism^33^. *In vitro* studies have demonstrated that 25(OH)D could inhibit adipogenesis and induce adipocyte apoptosis^34^. Assumptions exist that the active form of vitamin D manifests the ability to inhibit differentiation of pre-adipocytes by alteration of activity of the adipogenic transcription factors^35^. Moreover, reports on the favorable effect of vitamin D supplementation on the process of reduction in adipose tissue were also appeared^36^. Although these findings substantially support our study, it still remains unresolved to what extent vitamin D deficiency represents the cause of obesity.

Furthermore, several studies have shown that nutrition supply could induce alterations in DNA methylation, and then result in changes in biological phenotype^21^. A recent study found that maternal vitamin D deficiency during pregnancy resulted in insulin resistance in rat offspring, which was associated with inflammation and *Iκbα* methylation^37^. However, alterations of methylation may more often than not be a phenomenon that affects multiple genomic loci. The influence of aging and environmental influences such as nutrition supply seems to be on global methylation patterns, in turn exerting local effects on groups of genes^38^. Hence, the gradually accumulated methylation alterations may help to interpret the phenotypic difference appeared in adulthood of offspring for maternal vitamin D deficiency.

In summary, through the animal experiments, cell experiments, and further mechanism exploration based on epigenetics, we concluded that vitamin D deficiency during pregnancy may promote the proliferation and differentiation of pre-adipocytes, which may be associated with methylation alterations of genes, such as *Vldlr* and *Hif1α*, ultimately leading to offspring obesity. Further studies are warranted to validate our hypothesis and extend our findings.

## Methods

The study was approved by the institutional review board of Nanjing Maternity and Child Health Care Institute, and the methods were carried out in accordance with the approved guidelines.

A total of 43 pregnant Sprague-Dawley rats were used. All breeding animals were housed in incandescent light devoid of ultraviolet B radiation on a 12 h light/dark cycle (lights on 0600 h), at a constant temperature of 21 ± 2 °C and 60% relative humidity, with food and water provided ad libitum. Four week-old female rats were allocated to two dietary groups, with each group matched for body weight. One group received a vitamin D replete diet (1000 I.U/kg, Dyets Inc., PA, USA) throughout the study (Control group). The another group was fed a vitamin D deficient diet (0 I.U/kg, Dyets Inc., PA, USA) from 4 weeks of age until birth. From birth, all rats and their offspring consumed the vitamin D replete diet. All pregnant rats were weighed weekly, and serum samples were collected every two weeks in order to measure the 25(OH)D concentrations. Male offspring were selected for the following experiments. They were weighed weekly before 6 weeks of age, and every two weeks after 6 weeks of age.

### Vitamin D measurement

Serum concentrations of 25(OH)D were measured by using an *in vitro* diagnostic enzyme immunoassay kit OCTEIA 25-Hydroxy Vitamin D (Immunodiagnostic Systems, Boldon, United Kingdom) according to the manufacturer’s instructions. The reported analytic sensitivity of the immunoassay was 6.8–380 nmol/L.

### Biochemical analysis and metabolic activity evaluation for offspring rats

Six offspring rats at 14 weeks old were randomly selected from each group. Plasma concentrations of total cholesterol (TC), triglycerides (TG), high density lipoprotein (HDL), and low density lipoprotein (LDL) were measured using commercial kits (Asan Pharmaco Co., Seoul, Korea) after fasting for 12 h.

Other six offspring rats were randomly selected from each group for intraperitoneal glucose tolerance test (IPGTT) at each week of age. Rats were fasted for 16 h and injected intraperitoneally with a 20% glucose solution prepared in saline (2 mg/g body weight). Blood was drawn from the tail vein before and after the glucose injection (at 0, 15, 30, 60, 120 min) and blood glucose was measured using an Arkray glucometer (GLUCOCARD II Series Test Meter, Japan).

Other six offspring rats were randomly selected from each group when a significant difference in the offspring weight between the two groups appeared. The offspring rats of the two groups were individually placed in the PhenoMaster/LabMaster device (TSE Systems, Germany). Water intake, food intake, locomotor activity, 24 h oxygen consumption (VO_2_), carbon dioxide production (VCO_2_), and heat production were recorded over 24 h at 40 min intervals. The respiratory exchange rate (RER) was estimated by calculating the ratio of VCO_2_/VO_2_.

### Body fat monitoring for offspring rats

Six offspring rats from each group were selected for body fat monitoring and fasted 24 h prior to anesthetization with ether. Transverse micro-CT images of the waist from L3 to L6 were scanned using a Micro-CT scanner (SkyScan 1176, SkyScan Co., Belgium) with resolution of 35 μm, voltage of 100 kV, current of 100 μA, exposure of 474 ms, and rotation step (degree) of 0.500. Analysis of micro-CT images was performed using Nrecon software (SkyScan Co.). Total volume (TV) and adipose tissue volume (AV) of abdomen were detected, and the ratio of AV/TV was calculated.

### Pre-adipocyte preparation and adipogenesis Assay

The offspring rats aged 7 days were anesthetized with ether and retroperitoneal adipose tissue was excised through a sterile laparotomy procedure. The tissue sample was sliced into small pieces and washed with phosphate-buffered saline (PBS). The tissue pieces were then digested in PBS containing 1 mg/mL collagenase for 60 min at 37 °C. The cellular pellet was isolated via centrifugation (1000 rpm for 15 min) and suspended in DMEM medium supplemented with 10% FBS. The pre-adipocytes were plated in culture flask and incubated in a humidified 5% CO2 incubator. To evaluate the effects of vitamin D deficiency on adipogenesis, a real-time cell proliferation assay and oil red O staining were conducted to evaluate the capacity of proliferation and differentiation, respectively.

The real-time cell proliferation assay was conducted using the xCELLigence® impedance-based, label-free, real time cellular analysis system (RTCA) (ACEA Biosciences, San Diego CA, USA) according to the manufacturer’s instructions. The pre-adipocytes containing 6 × 10^4^ cells were seeded in each well of E-Plate L8. The plate was then inserted into the RTCA machine caged in incubator and the cell growth was continuously monitored for at least 72 h in 1 h intervals. Data was analyzed using the RTCA Software 1.2 program (Roche Diagnostics). All data is presented as the mean normalized cellular index over time. All xCELLigence experiments were performed in triplicates.

The pre-adipocytes were cultured in 6-well plates and maturation was induced. For oil red O staining, the culture medium was removed and cells were washed twice with PBS. Cells were fixed with 4% formalin/PBS for 30 min at room temperature. The fixation cells were washed with PBS again and stained with 0.5% Oil Red-O/isopropyl alcohol solution for 60 min at room temperature. After washing three times, cells were observed using an optical microscope and photographed.

### DNA methylation profiling

For VDD offspring and CTL offspring at 14 weeks old, a total of six DNA samples from adipose tissue were used to perform comprehensive DNA methylation profiling of gene promoters and CpG islands to determine the differentially methylated genes using methylated DNA immunoprecipitation followed by hybridization to the NimbleGen DNA Methylation Promoter Plus CpG Island Microarray. And RNA-seq was used to determine the differentially expressed genes. For further validate the methylation and expression levels of the differentially methylated and expressed genes, Sequenom EpiTYPER^®^ and reverse transcription-quantitative polymerase chain reaction (RT-qPCR) were used in another six DNA samples from adipose tissue of the two groups. The significant differentially methylated regions were defined as at least one CpG site showing absolute difference in methylation level of ≥50% between groups at a significance level of *P*-value ≤ 0.05. A FDR (False Discovery Rate) correction was also applied to correct for multiple hypothesis testing.

### Statistical analysis

All the data were presented as mean ± standard error (SE). The statistical significances between VDD and CTL group were performed using Student’s *t*-test. *P* ≤ 0.05 in a two-sided test was considered statistically significant and indicated in the figures.

## References

[1] Bass R, Eneli I (2015). Severe childhood obesity: an under-recognised and growing health problem. Postgrad Med J.

[2] McCrindle BW (2015). Cardiovascular consequences of childhood obesity. Can J Cardiol.

[3] Apovian CM (2013). The clinical and economic consequences of obesity. Am J Manag Care.

[4] McMillen IC (2008). Developmental origins of adult health and disease: the role of periconceptional and foetal nutrition. Basic Clin Pharmacol Toxicol.

[5] Yajnik CS (2008). Vitamin B12 and folate concentrations during pregnancy and insulin resistance in the offspring: the Pune Maternal Nutrition Study. Diabetologia.

[6] Mutt SJ, Hypponen E, Saarnio J, Jarvelin MR, Herzig KH (2014). Vitamin D and adipose tissue-more than storage. Front Physiol.

[7] Mizwicki MT, Menegaz D, Yaghmaei S, Henry HL, Norman AW (2010). A molecular description of ligand binding to the two overlapping binding pockets of the nuclear vitamin D receptor (VDR): structure-function implications. J Steroid Biochem Mol Biol.

[8] Christakos S, Ajibade DV, Dhawan P, Fechner AJ, Mady LJ (2010). Vitamin D: metabolism. Endocrinol Metab Clin North Am.

[9] Mulligan ML, Felton SK, Riek AE, Bernal-Mizrachi C (2010). Implications of vitamin D deficiency in pregnancy and lactation. Am J Obstet Gynecol.

[10] Triunfo S, Lanzone A (2016). Potential impact of maternal vitamin D status on obstetric well-being. J Endocrinol Invest.

[11] Hypponen E, Power C (2006). Vitamin D status and glucose homeostasis in the 1958 British birth cohort: the role of obesity. Diabetes Care.

[12] Arunabh S, Pollack S, Yeh J, Aloia JF (2003). Body fat content and 25-hydroxyvitamin D levels in healthy women. J Clin Endocrinol Metab.

[13] Blum M, Dallal GE, Dawson-Hughes B (2008). Body size and serum 25 hydroxy vitamin D response to oral supplements in healthy older adults. J Am Coll Nutr.

[14] Robinson C, Chiang M, Thompson SN, Sondike SB (2012). Occurrence of vitamin D deficiency in pediatric patients at high risk in West Virginia. South Med J.

[15] Zhu W (2013). Calcium plus vitamin D3 supplementation facilitated fat loss in overweight and obese college students with very-low calcium consumption: a randomized controlled trial. Nutr J.

[16] Cashman KD (2016). Vitamin D deficiency in Europe: pandemic?. Am J Clin Nutr.

[17] Tao M, Shao H, Gu J, Zhen Z (2012). Vitamin D status of pregnant women in Shanghai, China. J Matern Fetal Neonatal Med.

[18] Crozier SR (2012). Maternal vitamin D status in pregnancy is associated with adiposity in the offspring: findings from the Southampton Women’s Survey. Am J Clin Nutr.

[19] Morales E (2015). Deficit of vitamin D in pregnancy and growth and overweight in the offspring. Int J Obes (Lond).

[20] Wen J (2017). Association of maternal serum 25-hydroxyvitamin D concentrations in second and third trimester with risk of gestational diabetes and other pregnancy outcomes. Int J Obes (Lond).

[21] Godfrey KM (2011). Epigenetic gene promoter methylation at birth is associated with child’s later adiposity. Diabetes.

[22] Gluckman PD (2007). Metabolic plasticity during mammalian development is directionally dependent on early nutritional status. Proc Natl Acad Sci USA.

[23] Lillycrop KA, Phillips ES, Jackson AA, Hanson MA, Burdge GC (2005). Dietary protein restriction of pregnant rats induces and folic acid supplementation prevents epigenetic modification of hepatic gene expression in the offspring. J Nutr.

[24] Lillycrop KA (2007). Induction of altered epigenetic regulation of the hepatic glucocorticoid receptor in the offspring of rats fed a protein-restricted diet during pregnancy suggests that reduced DNA methyltransferase-1 expression is involved in impaired DNA methylation and changes in histone modifications. Br J Nutr.

[25] Nascimento FA, Ceciliano TC, Aguila MB, Mandarim-de-Lacerda CA (2012). Maternal vitamin D deficiency delays glomerular maturity in F1 and F2 offspring. PLoS One.

[26] Parikh SJ (2004). The relationship between obesity and serum 1,25-dihydroxy vitamin D concentrations in healthy adults. J Clin Endocrinol Metab.

[27] Carbone LD (2008). 25-Hydroxyvitamin D, cholesterol, and ultraviolet irradiation. Metabolism.

[28] Renzaho AM, Halliday JA, Nowson C (2011). Vitamin D, obesity, and obesity-related chronic disease among ethnic minorities: a systematic review. Nutrition.

[29] Zhang MX (2015). Vitamin D Deficiency Increases the Risk of Gestational Diabetes Mellitus: A Meta-Analysis of Observational Studies. Nutrients.

[30] Lu M, Xu Y, Lv L, Zhang M (2016). Association between vitamin D status and the risk of gestational diabetes mellitus: a meta-analysis. Arch Gynecol Obstet.

[31] Vanlint S (2013). Vitamin D and obesity. Nutrients.

[32] Mai XM, Chen Y, Camargo CA, Langhammer A (2012). Cross-sectional and prospective cohort study of serum 25-hydroxyvitamin D level and obesity in adults: the HUNT study. Am J Epidemiol.

[33] Wang J (2016). Genome-wide association analysis identifies variation in vitamin D receptor and other host factors influencing the gut microbiota. Nat Genet.

[34] Kong J, Li YC (2006). Molecular mechanism of 1,25-dihydroxyvitamin D3 inhibition of adipogenesis in 3T3-L1 cells. Am J Physiol Endocrinol Metab.

[35] Ding C, Gao D, Wilding J, Trayhurn P, Bing C (2012). Vitamin D signalling in adipose tissue. Br J Nutr.

[36] Rosenblum JL, Castro VM, Moore CE, Kaplan LM (2012). Calcium and vitamin D supplementation is associated with decreased abdominal visceral adipose tissue in overweight and obese adults. Am J Clin Nutr.

[37] Zhang H (2014). Maternal vitamin D deficiency during pregnancy results in insulin resistance in rat offspring, which is associated with inflammation and Ikappabalpha methylation. Diabetologia.

[38] Maier S, Olek A (2002). Diabetes: a candidate disease for efficient DNA methylation profiling. J Nutr.

